# Potential impact and challenges associated with Parkinson’s disease patient care amidst the COVID-19 global pandemic

**DOI:** 10.1186/s40734-020-00089-4

**Published:** 2020-08-08

**Authors:** Ali Elbeddini, Anthony To, Yasamin Tayefehchamani, Cindy Wen

**Affiliations:** 1 Leslie Dan Faculty of Pharmacy, University of Toronto, 144 College St, Toronto, M5S 3M2 Canada; 2 Winchester District Memorial Hospital, 566 Louise Street, Winchester, ON KK0C2K0 Canada

**Keywords:** COVID-19, Parkinson’s disease, Patient care, Telemedicine

## Abstract

**Background:**

COVID-19 has made itself known to health care providers and families across the world in a matter of months. While primarily a respiratory disorder, it has also been shown to cause neurological symptoms, which can be a concern for Parkinson’s disease (PD) patients. Although PD is not as common as other conditions such as cardiovascular diseases, it affects millions of patients around the world whose care has been affected by the global pandemic.

**Objectives:**

The aim of this review is to provide insight into the direct and indirect associations between COVID-19 and PD patient care.

**Results:**

Potential direct effects of COVID-19 include possible neurodegeneration, concerns of symptom self-management with over-the-counter (OTC) products and ICU challenges that can arise in PD patients. In addition, a subset of PD patients may be at higher risk of severe COVID-19 infection. The indirect effects of the pandemic are associated with the social distancing measures and disruptions in health care systems and PD clinical trials, which may negatively affect PD patients’ mental wellbeing and create barriers in controlling their PD symptoms. On a more positive note, telemedical care is quickly emerging as a primary communication tool for virtual patient care. However, further research should be conducted to examine the applicability of telemedicine across the entire PD population, such as those with more severe symptoms living in less developed areas. With all the uncertainty during this time, it is hopeful to hear many promising COVID-19 treatments being researched, one of them being a PD drug therapy, amantadine.

**Conclusion:**

Hopefully, we can consider this pandemic an opportunity to strengthen the PD community and learn more about the impact of the SARS-COV-2 virus. This review provides an overview of the interaction between COVID-19 and PD patients and future investigational retrospective studies are suggested to validate the observations.

## Background

The world is facing an unprecedented time where new patients are being infected by the novel coronavirus called severe acute respiratory syndrome coronavirus (SARS CoV-2). Starting as an epidemic in Wuhan, China at the beginning of December 2019, SARS-CoV-2 has spread so quickly that the World Health Organization officially called it ‘coronavirus disease 2019’ (COVID-19) and declared it a pandemic on March 11, 2020. COVID-19 has now spread to 216 countries and has over 6.9 million confirmed cases worldwide as of June 7, 2020 [1]. COVID-19 is mainly a respiratory disorder that causes most patients to be asymptomatic or present with mild upper respiratory symptoms such as fever, dry cough, sputum production, shortness of breath and sore throat. However, severe manifestations may also occur causing acute respiratory distress which may lead to death. Additionally, there have been reports of neurologic complications associated with COVID-19 as well. Undoubtedly, the COVID-19 pandemic has caused drastic changes to health care systems as well as new challenges to social life brought by social distancing and lockdown measures across the world.

**Fig. 1:**
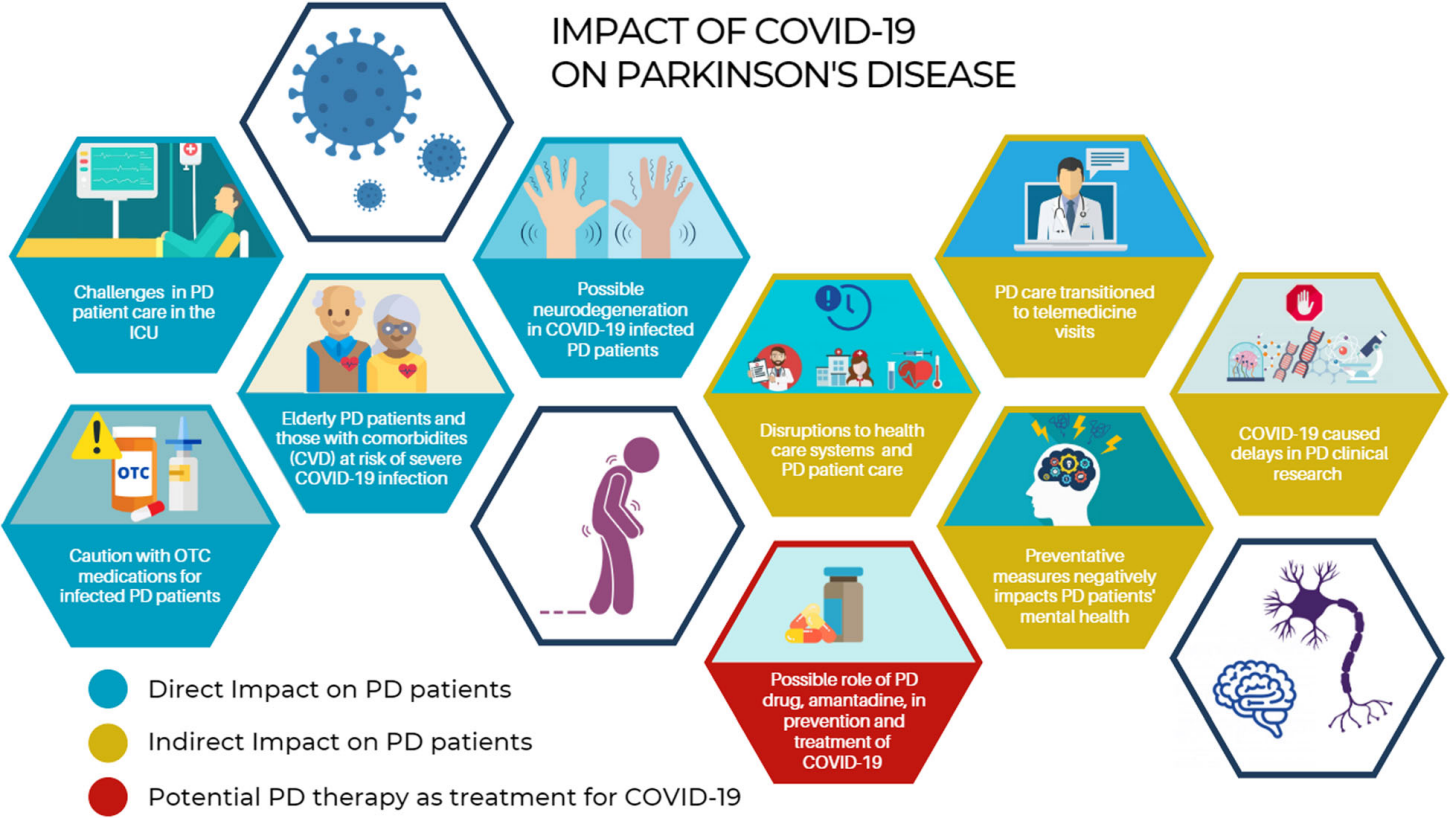
A concept map highlighting the key direct, indirect and drug therapy correlations between COVID-19 and PD patients

There are concerns that patients with health conditions are more vulnerable to the impact of COVID-19, including neurological conditions like Parkinson’s Disease (PD). PD is a chronic progressive neurodegenerative disease that manifests with key features including tremor, bradykinesia, and rigidity. Nonmotor symptoms including dementia, psychosis and autonomic dysfunction may present as the disease progresses. PD patients could be at higher risk of diseases as well as many PD patients are elderly and have multiple comorbidities. With PD affecting many individuals around the world, it is important to understand how they are impacted by the current pandemic. We conducted a literature search using the keywords “covid”, “coronavirus”, and “parkinson” and summarized key findings in this review. We also discuss the key direct and indirect interaction between COVID-19 and PD patients, as well as explore a promising COVID-19 treatment drug within the PD field (Fig. 1).

## Discussion

### Direct impact of COVID-19 on PD patients

#### Possible neurodegeneration among COVID-19 infected PD patients

The SARS-CoV-2 virus is of RNA origin and has a higher infectivity rate than the influenza virus [2]. Once infected, the virus glycoprotein can bind to angiotensin-converting enzyme 2 receptors (ACE2) which are highly expressed in the lungs [3]. This can result in acute alveolar damage, pulmonary edema and inflammation and evolve into acute respiratory distress syndrome (ARDS). Although COVID-19 mainly affects the respiratory system, there is evidence that SARS-CoV-2 infects the brain including the brainstem [4]. As PD is a neurological disorder, there is a concern that SARS-CoV-2 brain penetration could worsen symptomology of PD patients. For many years, antibodies against coronavirus have been found in the cerebrospinal fluid of PD patients, suggesting a role of viral infection in neurodegeneration [5]. Neurological manifestations associated with COVID-19 have also been well documented including dizziness, headache, hyposmia, hypogeusia, dysphagia and nerve pain [6]. Additionally, there is evidence that fever is associated with motor deterioration with PD patients and can even predispose them to parkinsonism-hyperpyrexia syndrome, a movement disorder emergency [6]. With fever being the most common symptom of COVID-19, as seen in 87.9% of affected patients, there is a strong possibility that COVID-19 can cause worsening of parkinsonian symptoms [6]. While there is no causal evidence that COVID-19 causes neurodegeneration, clinical case reports of worsening PD features have been documented in PD patients infected with COVID-19 [5]. In a case report of 8 PD patients, all showed worsened motor functions that lead to additional levodopa dosing [5]. Additionally, an observational case-control study found that motor symptoms significantly worsened in COVID-19 infected PD patients compared to noninfected PD controls [7]. Although, it was speculated that clinical changes may be caused by systemic inflammatory response rather than viral invasion of the central nervous system [7]. Nonmotor symptoms also appeared to be affected, however was not consistently described among different cases. Cilia et al. reported increased urinary urge/incontinence in infected patients [7], while Antonini et al. described worsened orthostatic hypotension, cognitive impairment and psychosis [5]. Both studies commonly observed increased fatigue in infected patients as well [5, 7]. Grabli and Hainque also highlighted the difficulty of detecting COVID-19 as some symptoms such as fatigue, asnomia, hot flush and painful limbs can also present as non-motor PD signs [8]. Current findings are suggestive of worsening motor and nonmotor PD symptoms following infection with COVID-19, however longitudinal studies would be beneficial to confirm this observation.

#### Concerns of mild COVID-19 symptom management with OTC medications in PD patients

Patients have been advised to manage their symptoms at home in most cases where COVID-19 infection appear to be mild of nature. Patients may present with symptoms of fever, dry cough, and sore throat with mild infection and many may choose to self-medicate with OTC products. For PD patients, it is especially important to discuss with a doctor or pharmacist about self-medicating since some OTC products can interfere with their Parkinson’s symptoms and medications. In addition to motor symptoms, PD patients may present with dementia as well as autonomic dysfunction such as bladder problems, constipation, sexual dysfunction, dry mouth, sweating and orthostatic hypotension [9]. Caution should be noted if PD patients take OTC medications containing antihistamines like diphenhydramine and dimenhydrinate, since they have anticholinergic properties and can worsen constipation, confusion as well as urinary symptoms. An additional concern is if PD patients are taking anticholinergic agents like benztropine or trihexyphenidyl for Parkinson’s symptoms. If these agents are taken together with OTC antihistamines, anticholinergic side effects like dry mouth, blurred vision, constipation, and urinary retention could be enhanced and therefore close monitoring is advised [10]. For PD patients taking monoamine oxidase B inhibitors (MAOI) like rasagiline, safinamide and selegiline, more serious drug-drug interactions can occur if taken together with cough syrups containing dextromethorphan or nasal decongestants containing pseudoephedrine, phenylephrine or phenylpropanolamine [11]. The combination of MAOIs and dextromethorphan is not recommended since MAOIs can enhance the serotonergic effects of dextromethorphan which can lead to serotonin syndrome [12]. If MAOIs and decongestants are taken together, it could enhance the alpha agonist effects of decongestants and lead to severe hypertensive outcomes as well [13]. As many OTC cough and cold medications are available as combination products, it is important to consult a pharmacist if deciding to self-medicate to avoid dangerous drug combinations and to safely treat mild COVID-19 symptoms.

#### Are PD patients at risk of COVID-19 infection?

The question of whether patients with PD are at greater risk of COVID-19 infection is of interest in the PD community. A case-control survey conducted in Italy aimed to investigate this matter. Fasano et al. found that the risk of COVID-19 infection did not differ between PD patients and the general population [14]. While the patients in the study were community-dwelling PD patients, the risk profile of severe patients living in nursing homes or long-term care facilities remains unclear. Regarding the risk of developing severe COVID-19 infections, reports have found that elderly patients and those with comorbidities, such as cardiovascular disease, are especially vulnerable to progression to severe COVID-19 infections [15]. To date, there is no evidence showing PD itself puts patients at higher risk of severe COVID-19 infections [16]. However, PD patients have been shown to have more comorbidities than patients without PD. In a large population study, patients with PD had more physical and nonphysical comorbidities than those without PD, namely coronary artery disease, cerebrovascular disease, and heart failure [17].PD prevalence also primarily affects the elderly, as the onset is usually around 65 to 70 years [18]. These comorbidities along with the older age of PD patients can increase their risk for more severe forms of COVID-19. There may also be a possible direct association with severe COVID-19 in a certain subset of PD patients. Older advanced PD patients with respiratory dysfunction may present with dyspnea, respiratory muscle rigidity and impaired cough reflex. These respiratory restrictions in PD patients put them at increased risk of pulmonary decompensation and pneumonia, which are features of severe COVID-19 infection [6].

#### Challenges faced by COVID-19 infected PD patients in the ICU

In the case that PD patients develop severe COVID-19 infection and require admission into the ICU, there are many issues that need to be considered. Severe respiratory issues such as acute respiratory distress syndrome and pneumonia secondary to COVID-19 may require patients to undergo ventilation. As noted earlier, PD patients may already have respiratory restrictions characterized by respiratory muscle bradykinesia, rigidity, and dystonia, which may make intubation more challenging [19]. Swallowing may also be negatively affected in these patients, where saliva can pool in the mouth and lead to aspiration [6, 19]. When coupled with weak coughs due to chest wall rigidity, there may be a higher risk of aspiration pneumonia as well, which can complicate COVID-19 management [6]. Although there is no published evidence supporting this association, it is worthwhile investigating in future retrospective studies. With respect to the care strategy for PD patients admitted to the ICU, there are no current guidelines [16]. However, efforts should be made to ensure PD patients continue to receive anti-PD therapy. In cases of pneumonia, maintenance of previous PD medications or an equivalent levodopa dose is crucial to avoid rigidity and further respiratory impairment from dopaminergic withdrawal [16]. Patients on apomorphine pump therapy and levodopa/carbidopa intestinal gel (LCIG) continuous infusion may be continued if already implemented. In some cases, PD therapy must be adapted in the ICU, such as in severely akinetic patients with dysphagia where oral administration of drugs is no longer possible. The easiest, most cost-effective and efficient way is to convert to levodopa solution which is given via a nasogastric tube [16]. Starting an apomorphine pump in the ICU is generally not advised, however can be considered only if akinesia poses a real risk to the patient [16]. Another option is to use transdermal rotigotine, however it is considerably less efficacious than levodopa or apomorphine [16]. Since there are no guidelines dictating the therapeutic alternative of choice to use in PD patients in the ICU, it may be determined using the medical team’s best judgement on a case-by-case basis.

### Indirect impact of COVID-19 on PD patients

#### Disruptions to global health care systems

The COVID-19 pandemic has certainly caused disruptions in health care systems which can have indirect effects on PD patients. Neurologists are essential in the circle of care for PD patients and it is important to think about how their impact from COVID-19 can have subsequent effects on patients. Like many health care providers, neurologists are at risk of exposure to COVID-19 patients and if infected, they will be restricted in their ability to provide care for PD patients. In some regions where there is a shortage of medical staff, some neurologists may also be forced to provide care for COVID-19 patients, which ultimately leads to less time spent caring for PD patients as well [16]. In many medical communities, nonurgent surgical procedures have been postponed to prevent patients from being infected. Regarding PD patients, elective surgical procedures like deep brain stimulation (DBS) have been delayed, as well as the initiation of LCIG and apomorphine pump [16]. These delays create barriers for PD patients from accessing vital medications that can control their condition, which can possibly lead to worsened symptoms. Fortunately, there has been no report so far on the impact of the pandemic on global medication transport and supply chain issues for PD patients [3].

#### The transition to virtual PD patient care

Another consequence of COVID-19 is the rapid implementation of telemedicine across many health care systems, whereby communication technology is used to provide virtual patient care. Many PD patients and neurologists have transitioned to telemedicine visits, particularly with synchronous videoconferencing. To assist movement disorders neurologists, the Movement Disorders Society (MDS) Telemedicine Study Group has created a step-by-step guide to implementing telemedicine [20]. The use of telemedicine and telerehabilitation to assess PD patients has been well documented and validated [21]. However, with virtual assessments, the Movement Disorder Society – Unified Parkinson’s Disease Rating Scale (MDS-UPDRS) can not be recommended since muscle rigidity and retropulsion pull testing can not be properly assessed via videoconferencing [16, 22]. Instead, a modified version of the MDS-UPDRS without rigidity and retropulsion pull testing is reliable and valid to use [23]. Advantages of telemedicine include access to specialists, convenience, time savings and cost reductions [24]. Nevertheless, there are still limitations to telemedicine. In a recent online survey with 781 PD patients who participated in telemedicine, the main concerns were lack of hands-on care, lack of intimacy and technical difficulties [25] A consistent barrier in providing virtual care is poor internet connectivity and video quality issues, which is especially limiting in less developed countries [24, 26]. To bypass this problem, asynchronous videos can be used to capture PD symptoms and sent to neurologists via email, which is more widely accessible [26].

In a review by Adams et al., several studies demonstrated that remote care of PD patients is feasible, effective, and valuable [24]. However, it is important to note the major limitations to these studies. The PD population may be underrepresented since participants had generally mild disease severity with UPDRS part III scores ranging from 24.2–44.1 (max score 128) and live in more developed countries like USA, Canada, Italy and Japan [24]. More severe patients with persistent tremor, rigidity and speech impairments may have a range of difficulties navigating telemedicine who were not represented in the studies. Future research involving more severe PD patients and those living in less developed countries is suggested to gain a more holistic understanding of the applicability of telemedicine in PD patient care.

There are also additional challenges with respect to virtual management of device aided therapies in PD, such as DBS and infusion pump devices, as telemedicine has not been universally established for these therapies [27]. Patients need to be educated on how to adjust device settings, monitor battery life, as well as troubleshoot device issues. With these new challenges in place, there is an emerging interest in the development of remote access to device programing, which could ease the technical burden on PD patients. Some pilot studies have been performed with remote control of apomorphine infusion where the results are promising [28]. In addition, 2 dB manufacturers, PINS Medical (Beijing, China) and SceneRay Corporation Ltd. (Suzhou, China) developed a remote, wireless DBS programming system where the settings may be adjusted remotely by clinicians [29–31]. Although this technology is currently only available in China, it is hopeful that it will become globally accessible soon.

#### Social distancing effects on PD patients’ mental health

The COVID-19 pandemic has also caused drastic changes to PD patients’ normal routine with social distancing and lockdowns in place across the world. Understandably, many PD patients will experience a negative impact on their mental health. In a case report from a movement disorder clinic in Cairo, PD patients reported worse stress, depression, anxiety and quality of life compared to matched controls during the pandemic [32]. A possible explanation is that the pathophysiology of PD naturally increases their risk of chronic stress since reduced dopamine levels impair coping mechanisms for stress [33]. This is concerning since stress can cause short term and long-term consequences for patients with PD. It has been shown that psychological stress can worsen motor symptoms such as tremor, gait and dyskinesia [34]. Stress can also reduce the effect of dopaminergic medications, such as levodopa’s effect on Parkinson’s tremor [35]. Increased stress may also unmask a latent hypokinetic rigid syndrome, perhaps leading to new PD diagnoses during the pandemic [6]. Preventative measures during the pandemic also significantly reduce mobility and physical exercise leading to a sedentary lifestyle. This is important to consider since physical exercise can attenuate clinical PD symptom progression and associated stress [33]. Thus, promoting home-based exercises, such as online exercise or dancing classes for PD patients, are crucial in maintaining their overall health during the pandemic.

#### Delays to novel PD drug therapies due to COVID-19

Another consequence that may be overlooked with respect to the COVID-19 pandemic is its impact on PD research and clinical trials. Many biopharmaceutical companies have delayed timelines for pipeline PD drugs due to COVID-19, which may result in additional burden on those whose parkinsonism is not adequately controlled by current medications on the market. We will discuss five pipeline medications in particular. First, Neurocrine Bioscience Inc’s novel drug ONGENTYS (opicapone) was recently approved by the FDA for add-on treatment to levodopa/carvidopa in PD patients experiencing “off” episodes, but will delay its launch in the US until later in 2020 due to COVID-19 [36]. ONGENTYS is an oral selective catechol-O-methyltransferase (COMT) inhibitor that blocks COMT from breaking down levodopa, resulting in more levodopa available to reach the brain and provide clinical effects [37]. Second, Addex Therapeutics has postponed the initiation of a Phase IIb/III clinical trial of dipraglurant, a novel orally available metabotropic glutamate receptor 5 (mGluR5) inhibitor, for PD patients with levodopa-induced dyskinesias [38]. Third, the enrollment for Phase 1 and Phase 1b trials have been paused for Denali Therapeutics’ backup pipeline drug DNL151 [39]. This small molecule inhibits LRRK2, which is an enzyme involved in lysosomal dysfunction and neurodegeneration, a key pathology seen in PD [39]. Fourth, a new investigational gene therapy drug, NBIb-1817 by Neurocine, has temporarily paused enrollment of patients into the Phase II RESTORE-1 Trial [40]. This novel therapy is aimed at delivering the aromatic l-amino acid decarboxylase (AADC) gene directly into neurons of the putamen, where AADC enzyme will be produced to convert levodopa into dopamine [40]. Fifth, resTORbio has also announced delays in enrollment of its fifth cohort in the ongoing Phase 1b/2a trial of RTB101 in PD patients [41]. RTB101 is a small molecule candidate that inhibits rapamycin complex 1 (TORC1), which contributes to the decline of neurologic function [42]. Unfortunately, these delays may negatively affect patients who are urgently waiting for new PD therapies to control their condition.

#### Potential PD therapy repurposed to treat COVID-19

The race to find a new vaccine or potential cure to COVID-19 has been on ever since the start of the pandemic. One investigational drug that is used to treat PD could be a possible prevention or treatment therapy for COVID-19 infection. Amantadine is an adamantine derivative that blocks NDMA activity. Its exact mechanism of action in PD is unknown, however it has a role in decreasing excess neuronal activity and neuroprotection [15]. Currently it is used as an add-on therapy to PD patients with persistent dyskinesia that is not controlled by existing therapy. Interestingly, amantadine also has antiviral activity, as it was initially marketed as treatment against influenza A [15]. Amantadine can block the matrix-2 (M2) protein ion channel, thereby inhibiting viral uncoating inside the infected cell [15]. In addition, there is a new model suggesting amantadine can disrupt lysosomal gene expression which could decrease the capacity of viral replication in COVID-19 [43]. Although it has yet to reach early clinical trials, its potential protective antiviral effects may be seen in various case reports. Rejdak et al. describes 15 neurological patients, 5 of whom have PD, who were receiving treatment with amantadine and were confirmed to have COVID-19 infection. All of them spent 2 weeks in quarantine and none had developed clinical manifestations of the virus [44]. These promising observations as well as its safe side effect profile warrants further studies for its use as a potential COVID-19 treatment.

## Conclusion

The COVID-19 pandemic has changed the lives of many around the world, causing respiratory distress and even death. PD patients, especially those that are elderly and have CVD comorbidities are likely at a higher risk of severe COVID-19 infection. Once infected, there may be concerns of neurodegeneration, issues with self-medication with OTC products as well as ICU challenges specific to PD patients. With social distancing and preventative measures in place, health care systems and clinical research have been disrupted, patient care has been transitioned to virtual means, and many patients’ mental health have been negatively affected. Amid all the uncertainty, it is encouraging to discover that a PD drug, amantadine, could be a potential protective and treatment for COVID-19. As the number of COVID-19 infected patients increases, it is imperative to learn more about the SARS-CoV-2 virus and its impact on different patient populations. With telemedicine emerging as a primary communication tool for PD patient visits, it is also important to realize its advantages and limitations. Further research should be considered in order to generalize its validity across the entire PD population. As noted in this review, patterns have been emerging with respect to PD patients, and future investigation should be performed to confirm the observations.

## Data Availability

Data sharing does not apply to this article as no datasets were generated or analyzed during the current study.
